# Spatial Heterogeneity of Metabolic Response to Drought Stress in *Medicago lupulina* L. Leaves

**DOI:** 10.3390/metabo16010080

**Published:** 2026-01-17

**Authors:** Xinglin Wang, Ning Lv, Yuyun Xu, Xingpan Meng, Yukun Jin, Hongbin Gao, Fei Li, Yin Yi, Lunxian Liu, Tie Shen

**Affiliations:** 1 Guizhou Provincial Leading Talent Workstation for Protein Design and Biological Imaging Innovation, Key Laboratory of National Forestry and Grassland Administration on Biodiversity Conservation in Karst Mountainous Areas of Southwestern China, School of Life Science, Guizhou Normal University, Guiyang 550001, China; 232100100407@gznu.edu.cn (X.W.); lvning@gznu.edu.cn (N.L.); 222100100438@gznu.edu.cn (X.M.); lifei2@gznu.edu.cn (F.L.); gzklppdr@gznu.edu.cn (Y.Y.); 2 School of Mathematical Sciences, Guizhou Normal University, Guiyang 550001, China; xuyun@gznu.edu.cn (Y.X.); lsamd@gznu.edu.cn (Y.J.); 3 Guizhou Key Laboratory of Advanced Computing, School of Cyber Science and Technology, Guizhou Normal University, Guiyang 550001, China; ghb@gznu.edu.cn

**Keywords:** drought stress, metabolomics, tricarboxylic acid (TCA) cycle, defense metabolism

## Abstract

**Background**: Drought stress is a primary environmental constraint limiting crop growth and productivity. Current drought-related plant research predominantly focuses on whole-leaf analyses, neglecting the spatial heterogeneity of metabolites within leaf tissues. **Methods**: This study combined transcriptomic and metabolomic approaches to investigate spatially distinct metabolic responses in marginal versus central regions of *Medicago lupulina* L. leaves under PEG-simulated drought. **Results**: Findings demonstrated that TCA cycle metabolites exhibited relative stability between leaf margins and centers under drought conditions, suggesting preserved core metabolic functionality in central tissues to sustain stress tolerance. Additionally, shikimic acid displayed a significantly reduced regional gradient in stressed tissues (PEG Margin vs. PEG Center) compared to controls. Phenylalanine, tryptophan, liquiritigenin, isoliquiritigenin, coproporphyrin III, and coproporphyrinogen III itself exhibited significantly increased internal gradient differences in stressed groups compared to control groups. The coordinated upregulation of key biosynthetic genes (e.g., TAT, AST, FNS II) in both the marginal and central regions of stressed leaves indicates a metabolic shift toward the biosynthesis of downstream defensive flavonoids. These metabolites and genes accumulated preferentially in margin regions of stressed leaves, indicative of localized activation of defense-associated metabolic pathways. **Conclusions**: This study reveals a spatially partitioned metabolic response to drought stress in *M. lupulina* leaves, where defensive metabolism is preferentially enhanced at the leaf margins while core metabolic homeostasis is maintained. These findings provide new spatial insights into plant drought acclimation and identify potential targets for improving crop resilience through the fine-tuning of local metabolism.

## 1. Introduction

When exposed to environmental abiotic stresses [1,2], plants activate defensive mechanisms that induce changes in secondary and primary metabolites, as well as complex metabolic regulatory responses. Such stresses significantly impact plant growth and productivity, and understanding abiotic stress responses is essential for improving plant tolerance to environmental challenges [3]. Drought, one of the most prevalent abiotic stresses [4], induces a series of plant responses. These responses include stomatal closure to minimize water loss [5], osmoprotectant accumulation to maintain cell turgor [6], the expression of drought-responsive genes [7], and the accumulation of stress-related proteins [8]. Collectively, these physiological and molecular adjustments constitute the key mechanisms through which plants enhance stress tolerance and adapt to dehydration [5,6]. Polyethylene glycol (PEG) treatment is widely employed as an experimental method to simulate the osmotic stress component of drought conditions [9]. This approach reproduces the initial water deficit signals experienced by plant roots under natural drought in a controlled laboratory environment [10]. As such, PEG-based treatments serve as a critical tool for studying early plant responses to osmotic stress, screening drought-tolerant germplasm, and elucidating drought-associated metabolic pathways.

Alfalfa, China’s predominant legume forage, produces over 90% of its biomass in arid or semi-arid regions [11]. However, water deficiency acts as a critical abiotic stressor, undermining yield consistency and crop quality in these areas and frequently resulting in severe economic repercussions [12]. Previous studies have investigated growth and metabolic response disparities in *Medicago* species under drought stress [13] and some have leveraged transcriptomics to explore molecular mechanisms governing stress responses [14,15]. For leaf-focused research, the majority have concentrated on comparative transcriptomic analyses of leaf tissues [16] assessments of two cultivated *Medicago sativa* L. varieties with divergent drought tolerances [17], and genome-wide studies of abiotic stress responses [18]. However, previous studies predominantly emphasize temporally averaged responses across entire leaf tissues under different stress durations [19,20], neglecting intra-leaf spatial heterogeneity in metabolic enrichment patterns.

Metabolites in leaves are not uniformly distributed [21] but exhibit specific accumulation patterns in different regions. The metabolite enrichment in distinct leaf zones (leaf margin and leaf center) under stress is intimately associated with key physiological processes and plant resilience mechanisms. This may represent a precisely regulated evolutionary strategy developed through plants’ long-term acclimation [22]. While stress-induced whole-leaf physiological and molecular responses have been well-documented [13,16], the differential enrichment of stress responses in distinct leaf tissue regions remains understudied and warrants further exploration. Previous studies have employed integrated transcriptomics and metabolomics to investigate the differences in enrichment between distinct colored regions of petals [23,24]. In a GC-MS analysis of salt-stressed barley seedling roots [25], regional specificity was observed in the coordination between root growth acclimation and metabolic pathways [25]. The precise spatial deployment of defensive compounds and optimal resource allocation directly influence plant environmental adaptability and the development of crop quality. For example, a recent study [26] developed a novel spatially resolved metabolomics approach, enabling microregional absolute quantification of 56 key metabolites in individual tea leaves. In *Camellia sinensis* (tea plant) leaves, compounds such as polyphenolic catechins and quercetin glycosides accumulated in specific locations following mechanical damage, suggesting their involvement in localized defense mechanisms.

Notably, *Medicago* a model legume species renowned for its cold and drought tolerance [27] and exceptional environmental adaptability [13], paradoxically faces escalating challenges to sustaining its productivity in modern agricultural systems. *Medicago lupulina* L. is widely distributed across Eurasia [27], grows widely in Karst habitat with strong tolerance to high calcium [28], and is commonly found in China. *M. lupulina* demonstrates robust drought tolerance [28] and is equipped with continuously expanding genomic databases and advanced genetic manipulation tools [29]. These resources enable precise molecular-level dissection of its drought response pathways, positioning it as an ideal model organism for elucidating mechanisms underlying abiotic stress (specifically drought stress). This species possesses diverse advantageous traits and broad application prospects, demonstrating critical value in forage production [30,31], ecological restoration [32], and other domains. Furthermore, it has emerged as a promising model system in applied ecology and plant stress physiology [33], showcasing its potential for advancing research into stress tolerance and sustainable agriculture.

Limited research has investigated differences in metabolic enrichment between different regions of a leaf, such as the margin versus the center. This study employed the model plant *M. lupulina* under simulated drought stress conditions. By integrating metabolomics and transcriptomics analyses via LC-MS, we compared metabolic fold changes between leaf margins and centers under drought stress (PEG Margin vs. PEG Center) and between their corresponding control regions (CK Margin vs. CK Center). Based on the hypothesis that leaf margins and central regions employ distinct strategies to cope with drought stress, this study aimed to identify key metabolites, genes, and their enriched regulatory pathways driving spatial heterogeneity in leaves under drought conditions. The findings are anticipated to provide a novel spatial perspective on plant drought acclimation mechanisms and identify potential targets for enhancing crop stress tolerance through precise modulation of local metabolic processes.

## 2. Materials and Methods

### 2.1. Materials 

The *Medicago lupulina* L. seeds used in this study were planted and cultivated in the growth chamber of the Key Laboratory of Life Science, Guizhou Normal University (altitude: 1180.0 m; latitude/longitude: 26°35′18″ N, 106°43′18″ E). The growth chamber was maintained under controlled conditions: 700 µmol photons m^−2^ s^−1^ light intensity, 50% relative humidity, 22 °C, and a 16-h-light/8-h-dark photoperiod. Seeds were treated with concentrated sulfuric acid for 2 min, followed by thorough rinsing with deionized water, a 24-h water soak. Select seedlings with uniform germination and transplant them into pots. For the first 30 days, plants were grown using half-strength Hoagland’s solution, with watering every 4 days. Control treatment: Half-strength nutrient solution (1/2 M). Drought treatment: Half-strength nutrient solution supplemented with 18% (*w*/*v*) PEG-8000. After 4 days of stress exposure, samples were collected. For sampling, two distinct leaf regions were dissected from both stressed and control plants: The marginal region is within 0.3 cm of the leaf margin. The central region is defined as the area surrounding the main vein. Leaves were categorized into four distinct groups: PEG Margin, PEG Center, Control Margin, and Control Center. Tissue samples were immediately immersed in liquid nitrogen for rapid preservation before downstream analyses.

### 2.2. Determination of Physiological Parameters

Photosynthetic measurements were conducted on sunny days between 09:00 and 11:00 h using an LI-6400XT portable photosynthesis system (LI-COR Biosciences, Lincoln, NE, USA), with net photosynthetic rate (Pn) as the key parameter. The instrument was warmed up for at least 20 min prior to use. The first fully expanded, healthy mature leaf from the top of each potted plant was measured. After enclosing the leaf in the cuvette, readings were recorded following a 2-min acclimation under set conditions. The leaf margin and center regions were measured separately using a localized shading method with aluminum foil. The cuvette conditions were set as follows: photosynthetic photon flux density at 1000 μmol m^−2^ s^−1^, flow rate at 500 μmol s^−1^, with the mixing fan activated to ensure uniform air flow. Leaf water potential (Ψ) was measured using a WP4-T dew point potentiometer (METER Group, Pullman, WA, USA). Fresh leaf discs from the four sample groups were immediately sealed in the instrument’s specialized sample cups, ensuring they were fully filled. The sealed cups were promptly placed in the measurement chamber for automatic analysis. Prior to measurement, the instrument was calibrated according to the manufacturer’s manual using standard salt solutions. Readings were recorded once stabilized, representing the leaf total water potential. Chlorophyll and carotenoid contents were quantified spectrophotometrically. Leaf segments (0.1 g fresh weight) from specific regions (avoiding the midvein) were homogenized in 10 mL of 80% (*v*/*v*) acetone and centrifuged at 13,813× *g* for 15 min. The extraction was repeated until the tissue became colorless. The absorbance of the supernatant was measured at 645, 663, and 470 nm using a UV-Vis spectrophotometer, with 80% acetone as a blank. Chlorophyll a, b, and total chlorophyll concentrations were calculated according to Arnon [34]. Carotenoid content was determined using the equations of Wellburn [35]. At least five independent biological replicates were analyzed per treatment.

Data are presented as the mean ± standard deviation (SD) of at least five independent biological replicates. Significant differences among the experimental groups were evaluated by analysis of variance (ANOVA), followed by Tukey’s honest significant difference (HSD) post hoc test for multiple comparisons. Differences were considered statistically significant at *p* < 0.05. The statistical analyses were performed, and graphs were generated to depict the mean values with SD error bars and annotate significant differences, using Origin 2025b (OriginLab Corporation, Northampton, MA, USA). for visualization.

### 2.3. Total RNA Extraction and High-Throughput Sequencing

Total RNA was extracted from 200 mg of plant tissue using TRIzol reagent (Invitrogen, Carlsbad, CA, USA) according to the manufacturer’s instructions. RNA purity and concentration were measured with a NanoDrop 2000 spectrophotometer (Thermo Fisher Scientific, Waltham, MA, USA), and RNA integrity was assessed using an Agilent 2100 Bioanalyzer (Agilent Technologies, Santa Clara, CA, USA). Transcriptome libraries were constructed using the VAHTS Universal V5 RNA-seq Library Prep Kit (Vazyme, Nanjing, China) following the provided instructions. Sequencing and data analysis were performed by Shanghai OE Biotech. Co., Ltd. (Shanghai, China).

### 2.4. Transcriptome Data Analysis and Differential Expression Gene (DEG) Profiling

Transcriptome sequencing was performed using the Illumina Novaseq 6000 [36] platform with paired-end reads of 150 bp. Library construction, sequencing, and preliminary data processing were conducted by Shanghai OBI Bio-Medical Technology Co., Ltd. (Shanghai, China). Raw reads in FASTQ format were filtered to obtain clean reads, which were subsequently assembled into transcripts de novo using Trinity [37]. The longest transcript for each gene was selected as the unigene for downstream analysis. Differential expression analysis was performed using DESeq2, with differentially expressed genes (DEGs) identified based on the criteria: adjusted *q*-value < 0.05 and |log_2_ (fold change)| ≥ 1 (fold change ≥ 2 or ≤0.5). Functional enrichment analysis of DEGs was conducted using R (version 3.2.0) for KEGG pathways and other biological processes. Principal component analysis (PCA) and hierarchical clustering were applied to visualize sample relationships, gene expression patterns, and enriched functional pathways. Additionally, the spatial gradient-specific effects of drought stress (simulated by PEG) on gene expression were further investigated to explore regional differences in transcriptomic responses.

To dissect the stress-specific alteration of gene expression gradients, we performed a stringent, two-tiered screening of the DEGs. First, we isolated a subset of genes showing significant expression disparity in PEG-treated tissues (margin vs. center). Subsequently, within this stress-responsive gene subset, we identified genes exhibiting significant alterations in leaf expression gradients under drought conditions. The selection criterion was a fold change exceeding 2-fold in the ratio of (PEG margin/PEG center) relative to (control margin/control center). This final candidate gene set captures transcripts underpinning differential spatial regulation in response to drought.

### 2.5. Metabolomics Profiling and Enrichment Analysis

Each group comprised six biological replicates. Sample Homogenization: 20 mg of tissue was weighed into 1.5 mL EP tubes, supplemented with two stainless steel beads, and mixed with 300 μL methanol (A452-4, HPLC grade, 99.9%)—water (*v*/*v* = 4:1, containing mixed internal standards). The tubes were pre-cooled for 2 min at −40 °C and subjected to mechanical grinding (45 Hz for 2 min) using a tissue lyser. After grinding, samples were subjected to ultrasonication in an ice-water bath for 30 min, followed by overnight incubation at −40 °C. Liquid-Phase Separation: Supernatants were collected by centrifugation at 13,813× *g* for 20 min at 4 °C, and 150 μL of the supernatant was transferred to LC-MS vials with insert sleeves for analysis. QC samples were prepared by pooling equal volumes of extracts from all samples, serving as technical replicates for instrument calibration. Metabolite separation and detection were performed using a Waters ACQUITY UPLC I-Class Plus system coupled with a Thermo Fisher Scientific QE HF hybrid quadrupole-orbitrap mass spectrometer (Waters, Milford, MA, USA; Thermo Fisher Scientific, Waltham, MA, USA). The chromatographic column, mobile phase composition, and analytical parameters were consistent with the protocol described in Meng et al. (2025) [38]. All reagents were pre-cooled to 4 °C before use. Metabolomics profiling was conducted by Shanghai OE Biotech. Co., Ltd. (Shanghai, China) (Table S1).

To precisely quantify the impact of drought stress on the spatial distribution patterns of metabolites, we calculated a spatial distribution change factor (FC) for each metabolite using the formula: FC = (PEG Margin/PEG Center)/(Control Margin/Control Center), where PEG Margin and PEG Center denote metabolite levels in leaf margins and central regions under PEG-induced drought stress, while Control Margin and Control Center represent corresponding levels in non-stressed controls. This FC value directly quantifies the extent to which drought stress alters the inherent spatial gradient of a metabolite’s distribution: FC ≥ 2 indicates that stress significantly enhanced the enrichment difference in the metabolite between the leaf margin and center; FC ≤ 0.5 indicates that stress significantly weakened this enrichment difference; and FC ≈ 1 suggests that its spatial distribution was not significantly perturbed. To evaluate statistical significance, Welch’s *t*-test (assuming unequal variances) was applied to compare stressed and control groups. Metabolites meeting both *p*-value < 0.05 and FC ≥ 2 or FC ≤ 0.5 were defined as spatially DAMs, representing those undergoing statistically significant and substantial spatial redistribution under drought stress.

To further elucidate the biological significance of DAMs, we performed pathway annotation using the KEGG database and conducted KEGG Enrichment Analysis to identify significantly enriched metabolic pathways. Revealed the core regulatory networks underlying spatial metabolic regulation in leaves under drought stress, providing insights into the metabolic reprogramming triggered by stress-specific spatial gradients.

## 3. Results

### 3.1. Spatial Variability in Physiological Indices Measurement

**Figure 1:**
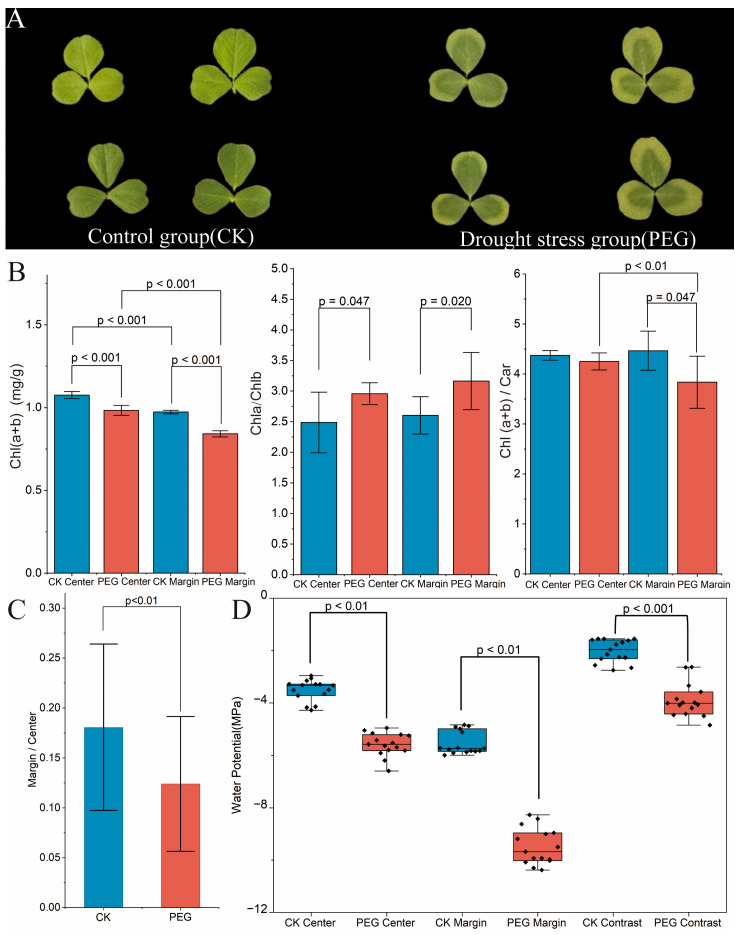
Physiological and phenotypic changes in *M. lupulina* leaves following 4 days of PEG8000-induced drought stress. (**A**) Leaf images show visible stress symptoms. (**B**) Altered pigment composition: total chlorophyll (Chl a + b), the Chl a/b ratio, and the Chl./Car. ratio. (**C**) Ratio of photosynthetic rate between the mid-leaf and leaf margin. (**D**) Leaf water potential. Diamond symbols represent data from individual biological replicate samples. CK represents the control group; PEG denotes the drought stress treatment group.

In response to drought stress, *M. lupulina* leaves developed a distinct chlorotic phenotype, initiating from the leaf margins and progressing inwards, which is indicative of preferential chlorophyll degradation in these regions (Figure 1A). This indicates a preferential degradation of chlorophyll in these areas (Figure 1B). Compared to control plants, both the center and margin tissues of stressed leaves showed a significant decrease in chlorophyll content, with the marginal regions experiencing a more pronounced decline. Additionally, the chlorophyll a/b ratio in the stressed leaf margin and central tissues was significantly higher than that in the control plants, while the chlorophyll-to-carotenoid ratio was significantly lower, especially in the marginal tissues. As shown in Figure 1C, drought stress caused a decline in overall photosynthetic capacity across the leaf, with marginal regions experiencing a far greater reduction than central tissues, reflected by a significant drop in the photosynthetic rate ratio (margin/center) under stress. Additionally, Figure 1D demonstrates that drought-induced water potential plummeted across the entire leaf, with marginal tissues undergoing preferential dehydration. Chlorosis at the leaf margin aligns with the observed decreases in chlorophyll ratio, photosynthetic efficiency, and water potential.

### 3.2. Transcriptomic Analysis

To investigate the transcriptional responses of *M. lupulina* leaves to drought stress under control versus polyethylene glycol 8000 (PEG8000) treatment, leaf samples were spatially partitioned into margin and central regions for transcriptome analysis. A total of 12 transcriptome sequencing libraries were constructed (Table 1), yielding 81.43 Gb of clean data. Each sample generated 5.86~7.05 Gb of clean reads with Q30 scores ranging from 94.65% to 95.17%, and an average GC content of 42.16%. De novo assembly identified 34,109 unigenes, with a total length of 46,517,580 bp and an average length of 1363.79 bp (Table 1). These results validate the high-quality assembly of transcriptomes.

**Figure 2:**
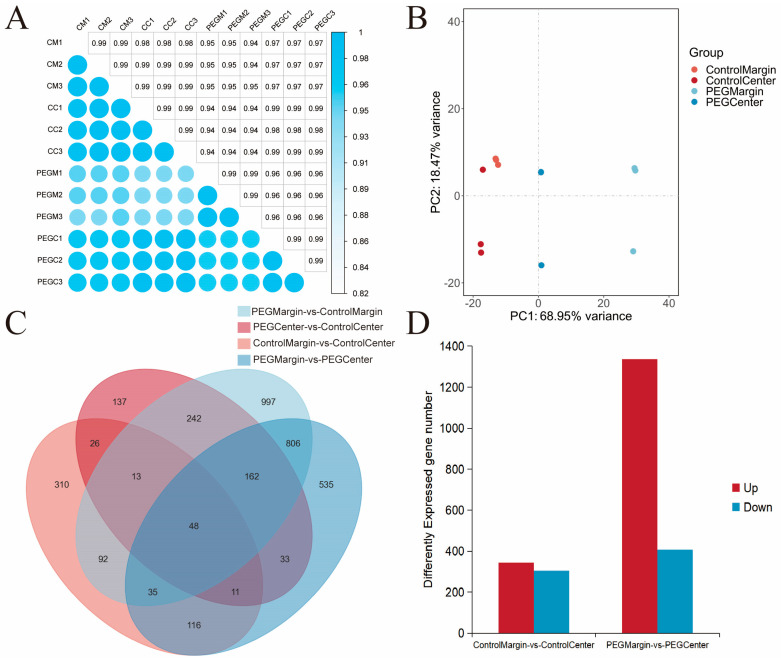
(**A**) Heatmap of pairwise correlation coefficients among samples. The *x*-axis and *y*-axis represent sample names, with color intensity indicating the magnitude of correlation coefficients. (**B**) Principal Component Analysis (PCA) plot. (**C**) Venn diagram illustrating shared and unique DEGs among comparison groups. (**D**) Bar chart depicting differential unigene expression statistics. The *x*-axis shows comparison groups, while the *y*-axis represents the total number of differentially expressed unigenes. “Up” denotes the count of significantly upregulated unigenes, and “Down” indicates significantly downregulated unigenes.

The biological replicates (Figure 2A) within both the control and stress groups exhibited exceptionally high consistency, with intra-group correlation coefficients of approximately 0.99, demonstrating excellent technical reproducibility. The correlation between Control Center and Control Margin was also around 0.99. Inter-group correlations between control and stress samples ranged from 0.94 to 0.96, indicating a maintained high positive correlation globally despite the treatment. Notably, stress-center samples showed consistently higher correlation coefficients with the control group than stress-margin samples did. Furthermore, the subtle but consistent difference between the PEG margin’s correlation with the control (0.94–0.96) and the correlation (~0.99) was both statistically and biologically significant. This pattern clearly reveals that the stress treatment induced subtle, gene-level changes. These changes allowed the treatment group to be distinguished from the control at the correlation level, with the PEG Margin region exhibiting the greatest divergence from both the PEG center and control groups. Based on the PCA results (PC1: 68.95%, PC2: 18.47%) (Figure 2B), the PCA plot revealed that PC1 and PC2 collectively explained 87.42% of the total variance. Control group samples (Center/Margin) clustered in the negative PC1 quadrant, while stress group samples (Center/Margin) occupied the non-negative PC1 region, demonstrating strong intra-group clustering. Within-group sample distributions were tightly clustered, reflecting robust experimental reproducibility. The Venn diagram (Figure 2C) illustrated the shared and unique DEGs among comparison groups. Notably, leaf margins and central regions in both stress and control groups displayed group-specific DEGs, indicating spatially distinct transcriptional responses. Figure 2D quantified the upregulated (Up) and downregulated (Down) DEGs across comparisons. The PEG Margin vs. PEG Center comparison exhibited the highest number of DEGs (1709, with a majority being upregulated), highlighting this as the most significant expression divergence. Conversely, the Control Margin vs. Control Center comparison showed minimal DEGs. Our analysis revealed that the stress treatment markedly accentuated the spatial heterogeneity of gene expression across the leaf blade.

### 3.3. Metabolomic Analysis

#### 3.3.1. Metabolomic Data Quality Assessment

This study employed liquid chromatography-mass spectrometry (LC-MS) for untargeted metabolomic profiling of the PEG Margin, PEG Center, Control Margin, and Control Center samples, identifying a total of 4161 metabolites. Metabolites were identified at confidence levels ≥ Level 2, ensuring the reliability of qualitative results. The relative abundances of these metabolites primarily included, Carboxylic Acids and Derivatives (12.26%), Fatty Acyls (14.92%), Organooxygen Compounds (10.45%), and Prenol Lipids (12.42%) (see Figure S1A).

PCA of the metabolomic data revealed distinct separation among stressed and control groups in leaf margins and central regions (Figure S2A), effectively capturing the primary sources of metabolic variation. PC1 predominantly distinguished stress-treated samples from control group samples, while PC2 further segregated samples based on leaf position (center vs. margin), highlighting inherent spatial differences in metabolite composition. The orthogonal partial least squares-discriminant analysis (OPLS-DA) plot (Figure S2B) demonstrated clear separation of all pairwise group comparisons, indicating significant specific differences. The OPLS-DA model effectively differentiated these groupings, further validating the specificity of the observed differences, which are both statistically significant (*p* < 0.05) and biologically interpretable.

#### 3.3.2. Comparative Analysis of Spatial-Specific Metabolic Responses: A Fold Change Perspective

**Figure 3:**
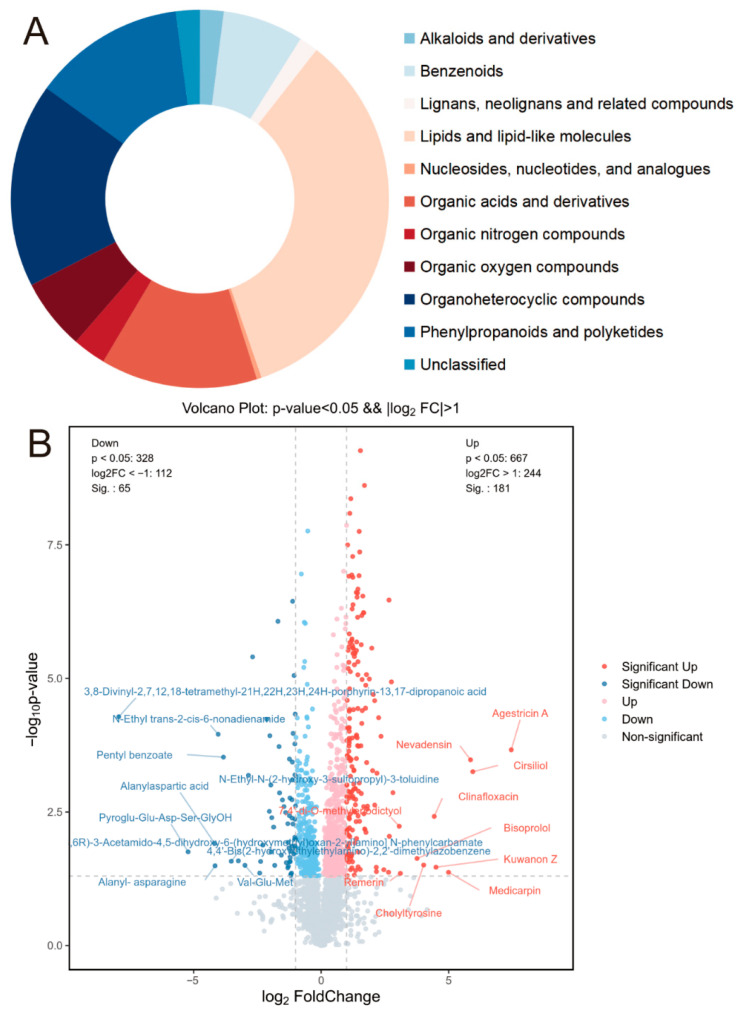
Metabolic profile and drought-induced spatial redistribution in *M. lupulina* leaves. (**A**) Pie chart showing the categorization of identified metabolites based on their biochemical classification. (**B**) Volcano plot displaying metabolites whose spatial distribution between leaf margin and center was altered by drought stress. The analysis compares the margin-to-center log_2_fold change in each metabolite under drought conditions against its corresponding margin-to-center log_2_ (fold-change) under well-watered (Control) conditions. Metabolites with significantly altered spatial patterns (*p* < 0.05 and |log_2_FC| ≥ 1) are highlighted in red (spatial difference enhanced by drought) or blue (spatial difference diminished by drought). Gray dots represent metabolites with non-significant changes in their spatial distribution. The comparison variants are: (PEG Margin vs. PEG Center) vs. (Control Margin vs. Control Center).

We integrated the metabolic gradient changes induced by stress in leaf tissues. Using screening criteria of *p* < 0.05, with fold change ≥2 or ≤0.5 (equivalent to log_2_FC ≥ 1 or log_2_FC ≤ −1), we identified 246 differentially accumulated metabolites (Figure 3A). Among these, 84 were lipids and lipid-related molecules, and 32 were phenylpropanoids and polyketides. Within the 246 metabolites, 65 were downregulated, and 181 were upregulated under stress conditions. Notably, lipids, lipid-related molecules, phenylpropanoids, and polyketides predominantly exhibited significant changes in response to stress, suggesting their critical roles in stress acclimation. The selected metabolites are displayed as a volcano plot (Figure 3B). “Up” denotes that the comparison between drought-stressed and control groups amplified the difference in metabolite levels between marginal and central regions under drought conditions. Conversely, “Down” indicates that the drought stress weakened the metabolic margin-to-center gradient in stressed leaves.

### 3.4. Combined Analysis of Transcriptomics and Metabolomics

#### 3.4.1. The Tricarboxylic Acid (TCA) Cycle Remains Stable Across the Leaf Gradient Under Stress

**Figure 4:**
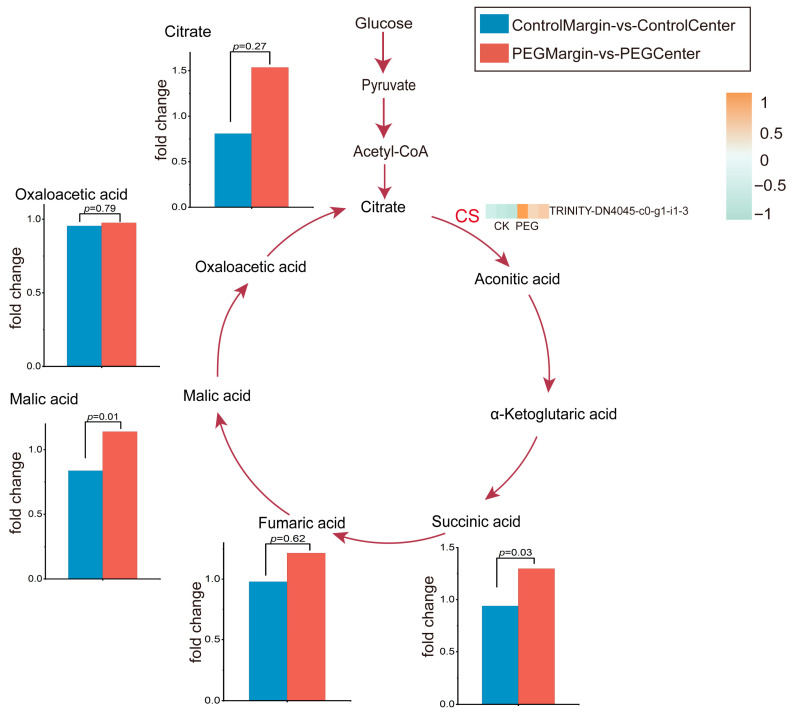
TCA Cycle Metabolic Pathway. The bar charts show the fold change differences between PEG Margin vs. PEG Center (drought-stressed leaf regions) and Control Margin vs. Control Center (non-stressed regions). Differentially expressed genes (DEGs) are indicated in red font. CK (Control Margin vs. Center Unigenes); PEG (PEG Margin vs. Center Unigenes). Color intensity indicates relative expression level.

TCA cycle serves as a central hub of cellular metabolism, responsible for energy production and providing carbon skeletons for biosynthesis. To evaluate its stability across the spatial gradient of leaf tissues under stress, we analyzed the spatial distribution change factor (FC) of core TCA intermediate metabolites (Table S2). Results showed that despite evident physiological damage in leaf margins under drought stress, the FC of the majority of TCA cycle intermediates remained close to 1 (Figure 4). Specifically: No significant change in the spatial distribution of fumaric acid and oxaloacetate was observed between regions (*p* > 0.05), as their fold changes showed no marked difference compared to the control group. Malic acid and succinic acid, although showing statistically significant *p*-values (*p* < 0.05), displayed minimal absolute FC changes (Malic acid FC = 1.36, succinic acid FC = 1.38), failing to meet the predefined threshold for spatial differential accumulation (FC ≥ 2 or ≤0.5). Citric acid exhibited a notable fold change (FC = 1.8), it failed to meet the screening criterion of FC ≥ 2 and lacked statistical significance due to excessively high intra-group variability. From a spatial perspective of “margin-to-center” distribution gradients, drought stress did not induce large-scale, directional spatial redistribution of core TCA intermediates.

#### 3.4.2. PEG-Stress Activates Defense-Related Metabolic Pathways

**Figure 5:**
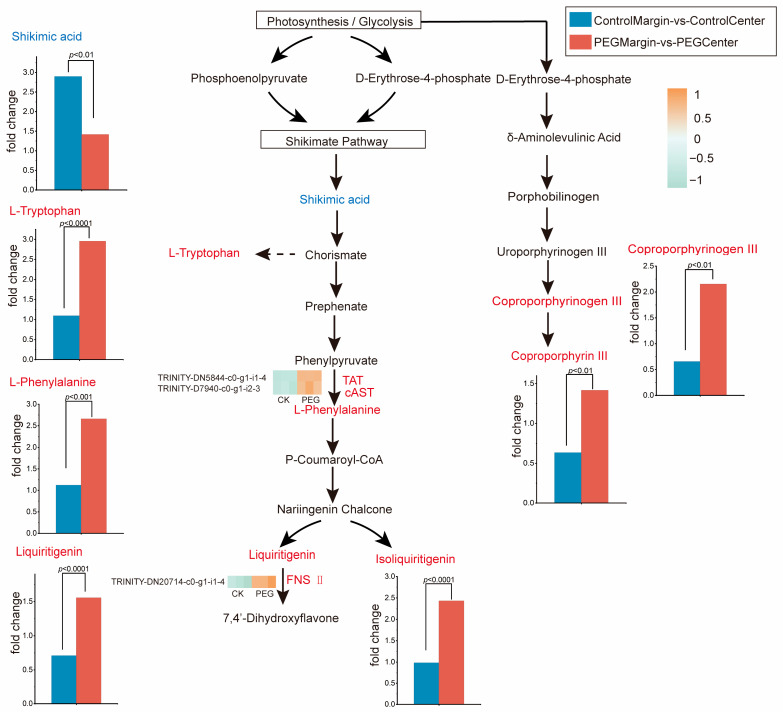
Comparison of Spatial Metabolite Distribution Differences Between Stress and Control Conditions. The bar charts show the fold change differences between PEG Margin vs. PEG Center and Control Margin vs. Control Center. Differentially expressed genes (DEGs) are indicated in red font. CK (Control Margin vs. Center Unigenes); PEG (PEG Margin vs. Center Unigenes). Color intensity indicates relative expression level. Dashed lines denote a multistep pathway for the substance’s formation.

Through integrative analysis of transcriptomic and metabolomic data, we identified stress-responsive differentially expressed genes (DEGs) and differentially accumulated metabolites, and pinpointed key enriched pathways of differential metabolites (Figure 5): the phenylalanine, tyrosine, and tryptophan biosynthesis pathway, the flavonoid biosynthesis pathway, and the porphyrin metabolism pathway (Figure S3A). KEGG enrichment analysis (Figure S4) revealed that transcriptomic responses were predominantly concentrated in amino acid degradation and stress signaling pathways. To further investigate direct associations with metabolite accumulation, we mapped the selected DEGs onto the three key enriched metabolic pathways of differentially accumulated metabolites. Metabolite profiling revealed that shikimate, a precursor of multiple secondary metabolites, exhibited a significant downregulation in the ratio of leaf margin-to-leaf center levels in stress-treated samples compared to controls (fold changes ≤ 0.5). This indicates that stress treatment reduced spatial distribution differences in shikimate between leaf margins and central regions. In stark contrast, its downstream products, phenylalanine and tryptophan, showed opposite trends with significantly upregulated relative ratios in stress-treated groups (fold changes ≥ 2). Transcriptomic analysis revealed that genes of TAT, AST, and FNSII were significantly upregulated exclusively in the margin/center regions of stress-treated leaves (PEG Margin/PEG Center). Notably, the upregulation of these genes in leaf margins and centers under drought stress was consistent with the elevated expression of phenylalanine and liquiritigenin. Phenylalanine, a key precursor for flavonoids and isoflavonoids, likely channels increased metabolic flux toward the synthesis of specific flavonoid defense compounds such as liquiritigenin and isoliquiritigenin (fold changes ≥ 2). These compounds act not only as direct antioxidants but also as intermediates for synthesizing diverse defense-related metabolites. In the Porphyrin metabolism, coproporphyrinogen III and coproporphyrin III were significantly elevated (fold changes ≥ 2) (Figure S3B) (Table S3). This further confirms that the tricarboxylic acid (TCA) cycle, acting as a central metabolic node, sees its downstream network extensively activated under stress. This coordinated activation facilitates various defense responses.

## 4. Discussion

To investigate regional metabolic differences within leaf tissues, we applied PEG-8000 (polyethylene glycol) to simulate drought stress in *M. lupulina* plants. PEG-8000, a non-invasive osmotic agent, reliably reduces external water potential to mimic soil water deficit without direct toxic effects. By integrating transcriptomic and metabolomic datasets, we found that under PEG-induced drought conditions, the metabolic differences in the TCA cycle between leaf margins and central regions remained stable. In contrast, defense-related metabolic pathways, such as phenylpropanoid and porphyrin metabolism, were significantly upregulated and exhibited spatial enrichment, particularly in marginal regions of the leaf.

### 4.1. Under Stress Conditions, the TCA Cycle Prioritizes Survival in Leaf Tissues by Maintaining Metabolic Stability

Previous studies predominantly employed whole-leaf homogenization to investigate stress-induced metabolic changes, such as the accumulation of amino acids and other metabolites across entire leaves. For instance, a study in the model plant Arabidopsis thaliana using non-targeted metabolomics [39] revealed significant increases in amino acids and organic acids under drought stress. The use of spatial sampling methods differs from previous studies employing whole-leaf homogenization techniques, which could only measure the average metabolic concentrations of entire leaves [40]. Whole-leaf homogenization may mask the spatial heterogeneity of metabolic changes between leaf margins and centers. The TCA cycle data from our study suggest that stress may not extensively reorganize the spatial distribution patterns of a subset of metabolites in the tricarboxylic acid cycle in leaf tissues. The spatially resolved analysis in this study provides direct evidence for this finding. We hypothesize that stress-induced metabolic changes might be rapidly coordinated across leaf regions via vascular tissues such as the phloem, thereby preserving spatial homogeneity. Notably, such spatial coordination and reallocation phenomena may represent a general mechanism. A study on pea (Pisum sativum) [41] further demonstrated that metabolomic changes in vascular tissues under drought stress reflect plant stress responses, highlighting their role as a key pathway for rapid transport of metabolites and signaling molecules. During stress signal transmission, vascular tissues facilitate inter-organ communication and resource reallocation by modulating metabolite flux in sieve elements [41]. Under resource-limited conditions, plants may prioritize essential physiological processes and balance stress responses with repair demands, maintaining TCA cycle stability across leaf regions to sustain survival and stress tolerance. Under drought stress, leaf margins and central regions adopt strategies to maintain functional stability of the TCA cycle, ensuring sustained energy supply and metabolic precursor availability.

Citrate synthase (CS; EC 4.1.3.7), the first and rate-limiting enzyme of the TCA cycle, catalyzes the condensation of acetyl-CoA and oxaloacetate to form citrate and CoA [42]. Functioning as the TCA pathway gatekeeper, CS constitutes a major regulatory node in central metabolism [43]. In this study, within the TCA pathway analyzed in Figure 4, we observed that stress did not significantly alter the regional distribution of TCA cycle metabolites. However, citrate synthase (CS) gene expression exhibited marked upregulation in leaf margins relative to central regions, specifically under drought stress. This margin-specific transcriptional induction aligns with the upstream regulatory hypothesis, where early transcriptional changes provide a template for subsequent metabolic adjustments [44]. Leaf margins, being more exposed to environmental stress—a phenomenon consistent with leaf chlorosis—under drought conditions, the TCA cycle metabolites in leaf centers adopt a response strategy prioritizing maintenance of network flux and functional homeostasis across spatial distributions. During the initial stress phase, plants perceive stress signals and upregulate transcription of the key enzyme citrate synthase (CS) gene, thereby enhancing mRNA and enzyme protein synthesis to prepare for escalating stress challenges. Studies in Arabidopsis thaliana under drought stress [45] revealed that leaf apex and margin regions initiate senescence programs at the transcriptional level, with visible leaf senescence observed in these areas following stress exposure. We hypothesize that this phenomenon reflects a transcriptional preadaptation mechanism. This strategy enables plants to maintain transient metabolic homeostasis while simultaneously preparing them to cope with potential, more severe stress episodes. Upon sensing initial stress signals, plants upregulate transcription of stress-responsive genes to preemptively synthesize enzyme proteins. This preadaptation enables immediate enzyme deployment when stress intensifies, rapidly enhancing the TCA cycle (tricarboxylic acid cycle) flux while bypassing the time lag of de novo transcription and translation. However, the prompt translation of transcriptional upregulation into metabolite accumulation risks over-accumulation of intermediate metabolites. Regulatory mechanisms maintaining homeostasis are therefore critical to mitigate these risks and ensure metabolic stability under fluctuating stress conditions.

### 4.2. Defense Metabolites Differentially Accumulate in the Margin Versus the Center Under Stress

As described above, the tricarboxylic acid (TCA) cycle exhibits relative stability across the spatial gradient of the leaf. Additionally, we identified defensive metabolic pathways specifically activated along this spatial axis, including Phenylalanine, tyrosine, and tryptophan biosynthesis, Shikimate metabolism, and Porphyrin metabolism. These metabolites exhibited significant alterations under stress conditions, with marked differences observed between PEG Margin/PEG Center and their corresponding Control Margin/Control Center comparisons. Research indicates [40] that plants activate conserved defensive metabolic programs in response to varying degrees of water deficit, which constitute central components of their stress response and survival strategies. For instance, transcriptome-metabolome integrated analyses revealed that phenylpropanoid biosynthesis and flavonoid biosynthesis play critical roles in mitigating drought stress in *C. lancifolius* seedlings [46]. Studies on *M. lupulina* drought responses further demonstrated that drought conditions directed the induction of biosynthetic pathways for multiple protective metabolites, including polyamines, flavonoids, phenolic acids, and coumarins [20]. Leaf margins are the first and most intensively exposed to environmental stress pressures, as evidenced by significant declines in photosynthetic performance, accelerated accumulation of ROS, and rapid water loss [45]. These findings are consistent with our physiological measurements. The phenylalanine, tyrosine, and tryptophan biosynthesis, Flavonoid biosynthesis, and porphyrin metabolism pathways are among the most critical metabolic processes in plants. In studies of drought stress, metabolites such as phenylalanine, tryptophan, liquiritigenin, isoliquiritigenin, coproporphyrinogen III, and coproporphyrin III were significantly upregulated [47]. In this study, phenylalanine and tryptophan exhibited significantly higher regional enrichment levels in the stressed groups (PEG Margin/PEG Center) compared to the control (Control Margin/Control Center). Previous studies have demonstrated that aminotransferase genes (TAT, AST, etc.) play a critical role in enhancing plant stress tolerance and are essential for plant growth and development [48]. Drought and low nitrogen stresses significantly elevate TAT activity in the roots of *Populus simonii* [49]. Furthermore, the involvement of TAT genes in the biosynthesis of phenylalanine, tryptophan, and tryptophan has been confirmed [48]. Transcriptomic data revealed that TAT and AST were significantly upregulated in the leaf margin/center regions (PEG Margin/PEG Center) of stressed groups, while no significant differences were observed in corresponding control regions (Control Margin/Control Center). The elevated expression of these genes in leaf margins and centers under drought stress corroborates their spatial correlation with increased phenylalanine and tryptophan levels. Phenylalanine, a precursor for secondary metabolites such as lignin and flavonoids, plays a key physiological role [50]. Phenylalanine contributes to reducing harmful ROS [51]. This spatial pattern of accumulation may reflect a mechanism to preferentially secure substrate supply for associated protective pathways in the vulnerable marginal region under stress.

Flavonoid biosynthesis in plants originates from the phenylalanine metabolic pathway [52,53]. Our findings revealed that the phenylalanine ratio between leaf margins and leaf centers in stressed tissues was significantly higher than in control groups, which may explain the increased accumulation of flavonoid metabolites, such as liquiritigenin and isoliquiritigenin, in stressed leaf margins. Previous studies demonstrated that under drought stress, the contents of isoliquiritigenin and liquiritigenin in licorice significantly increase, functioning as effective antioxidants [52]. During drought conditions, plants produce excessive ROS, leading to oxidative damage [54]. As a defense response, plants activate their antioxidant systems. In our study, liquiritigenin and isoliquiritigenin levels in stressed leaf margins were not only higher than those in central regions of the same stressed leaves but also significantly elevated compared to unchallenged control leaves. Under drought stress, the significant accumulation of liquiritigenin—the direct substrate for FNS II—in the leaf margins may trigger a substrate-induced or feedback-activation mechanism, upregulating the expression or activity of FNS II. This enables cells to efficiently utilize the available substrate pool and facilitates the rapid conversion of liquiritigenin into downstream flavones [55], such as apigenin and luteolin, particularly as stress intensifies. This spatial distribution pattern suggests that under stress, plants may prioritize the activation of secondary metabolic pathways in leaf margins to synthesize increased levels of antioxidant flavonoids.

Shikimic acid serves as a precursor for both phenylalanine and tryptophan. The accumulation of coproporphyrinogen III and coproporphyrin III may reflect metabolic adjustments in margin cells responding to chloroplast damage and repair processes under stress conditions. Previous studies have demonstrated that coproporphyrinogen III levels continuously increase under light stress [56]. In *Nitraria tangutorum* [57], drought stress activates the tetrapyrrole metabolism pathway, where one branch upregulates protoporphyrin IX synthesis (a chlorophyll precursor), while another branch specifically enhances coproporphyrin III production. In our study, coproporphyrinogen III and coproporphyrin III exhibited significantly higher margin-to-center gradients in stressed leaves compared to controls [57]. We speculate that coproporphyrin III may compete with coproporphyrinogen III for downstream chlorophyll biosynthetic pathways, potentially impairing chlorophyll synthesis and accelerating leaf margin yellowing. This hypothesis aligns with the observed early chlorosis in leaf margins, which coincides with the preferential accumulation of these porphyrin intermediates in stressed margin regions.

This study demonstrates significant spatial heterogeneity in leaf metabolism under drought stress. Drought-induced leaf chlorosis (Figure 1) is likely linked to internal water and light gradients within the leaf, which are further intensified under stress conditions. Leaf marginal tissues exhibited higher chlorophyll a/b ratios and lower chlorophyll to carotenoid ratios (Figure 1), indicating a light-protective pigment reorganization characterized by chlorophyll degradation and carotenoid accumulation. This phenomenon is plausibly triggered by the more pronounced decline in water potential at leaf margin. Metabolomic and transcriptomic analyses reveal that TCA cycle intermediates remain stable in central leaf regions (Figure 4), whereas leaf margins specifically accumulate multiple defense-related metabolites (Figure 5). While physiological, transcriptomic, and metabolomic data strongly support these interpretations, quantification of dynamic changes in cellular morphology and subcellular structures under stress remains unexplored. Future research could integrate spatial metabolomics with high-resolution cellular imaging to directly link metabolite profiles to physical states of cells. Additionally, validating differentially expressed genes via real-time fluorescent quantitative PCR (qRT-PCR) would further consolidate our findings at the transcriptional level, systematically elucidating the causal mechanisms underlying spatial metabolic strategies in leaves.

## 5. Conclusions

This study investigated regional metabolic differences between leaf margins and centers under drought stress condition and provided a novel perspective on the spatial heterogeneity of leaf metabolism during stress. TCA cycle intermediates (e.g., fumaric acid, Oxaloacetate) exhibited no significant regional differences between leaf centers and margins under stress, suggesting their stability in maintaining foundational energy and carbon skeleton supply. However, defense-related metabolites in downstream pathways, including flavonoids, phenylpropanoids, and porphyrins, showed marked margin-specific enrichment in stressed leaf margins compared to central regions. In leaves, while photosynthesis provides most of the energy, the TCA cycle in respiration remains critically important. This study found that the photosynthetic rate at the leaf margins is lower than that in the central region, with this discrepancy exacerbated under drought stress. Despite the drought, the TCA cycle persists in maintaining stability, likely to ensure continuous supply of energy and metabolic precursors, fulfilling the basic cellular demands for stress acclimation and survival. Shikimic acid serves as a precursor for phenylalanine and tryptophan biosynthesis. Under stress conditions, phenylalanine and tryptophan are margin-enriched in leaf margins, responding to the imposed stress. Flavonoid metabolites (e.g., liquiritigenin/isoliquiritigenin) exhibit stress-induced regional variations. PEG margin of coproporphyrinogen III and coproporphyrin III in leaf margins likely reflects chloroplast photodamage and repair processes under stress. Overall, plants may adopt a “two-tiered” metabolic strategy: while globally activating defense synthesis pathways to combat stress, they simultaneously maintain spatial stability in foundational energy metabolic networks (e.g., TCA cycle) across leaf tissues, ensuring core physiological functions remain balanced despite regional stress exposure.

## Figures and Tables

**Table 1:** RNA sequencing data quality.

Sample	RawReads(M)	RawBases(G)	CleanReads(M)	CleanBases(G)	ValidBases(%)	Q30(%)	GC(%)
ControlCenter1	23.82	6.91	22.83	6.62	95.83	94.9	42.26
ControlCenter2	25.36	7.3	24.12	6.95	95.09	94.99	42.17
ControlCenter3	24.95	7.27	24.01	6.99	96.23	95.17	42.2
ControlMargin1	24.55	7.16	23.64	6.89	96.3	95.11	42.36
ControlMargin2	25.64	7.38	24.35	7	94.95	94.82	42.26
ControlMargin3	25.27	7.33	24.21	7.03	95.83	95.02	42.4
PEG Center1	25.02	7.22	23.83	6.87	95.21	94.82	42.27
PEG Center2	27.37	7.65	25.24	7.05	92.22	94.65	42.15
PEG Center3	23.22	6.73	22.24	6.45	95.78	95.01	42.06
PEG Margin 1	22.36	6.3	20.8	5.86	93.01	94.79	41.85
PEG Margin 2	24.22	7.02	23.19	6.73	95.74	95.1	41.89
PEG Margin 3	25.59	7.36	24.29	6.99	94.95	94.93	42.06

## Data Availability

The raw data supporting the conclusions of this article will be made available by the corresponding authors on request. Data are available from the manuscript and Supplementary Files.

## Supplementary Materials

The following supporting information can be downloaded at: https://www.mdpi.com/article/10.3390/metabo16010080/s1, Table S1. Metabolomics Matrix; Figure S1: Pie Chart of Metabolite Class Distribution.; Figure S2: Principal Component Analysis (PCA) and Orthogonal Projections to Latent Structures Discriminant Analysis (OPLS-DA); Table S2. Fold Changes and Significance Analysis of TCA Cycle Metabolites; Figure S3. Overview and clustering analysis of pathway-enriched metabolites; Figure S4. Pathway enrichment analysis of differentially expressed genes; Table S3. Changes in Metabolite Levels and Significance Analysis in Defense Metabolic.

## Abbreviations

The following abbreviations are used in this manuscript:

UPLC–MS/MS	Ultra-High-Performance Liquid Chromatography–Tandem Mass Spectrometry
Car	Carotenoid
Chl	Chlorophyll
DAMs	Differentially Accumulated Metabolites
DEGs	Differentially Expressed Genes
FDR	False Discovery Rate
FC	Fold Change
HMDB	Human Metabolome Database
KEGG	Kyoto Encyclopedia of Genes and Genomes
log_2_FC	log_2_ Fold Change
OPLS-DA	Orthogonal Partial Least Squares-Discriminant Analysis
PAL	Phenylalanine Ammonia-Lyase
PCA	Principal Component Analysis
PEG-8000	Polyethylene glycol-8000
QE HF	Quadrupole-Orbitrap HF
RNA-seq	RNA Sequencing
TCA	tricarboxylic acid cycle
TRIzol	Tri Reagent
VAHTS	Vazyme Advanced High-throughput Sequencing
Welch’s *t*-test	Welch’s Student’s *t*-test
