# Supplementary material for: Spatial Heterogeneity of Metabolic Response to Drought Stress in Medicago lupulina L. Leaves
Source: Metabolites. 2026 Jan 17;16(1):80. doi: 10.3390/metabo16010080 (PMC12844375; doi:10.3390/metabo16010080)
Supplement: Supplementary file 1 [file metabolites-16-00080-s001.zip › metabolites--supplementary.pdf]

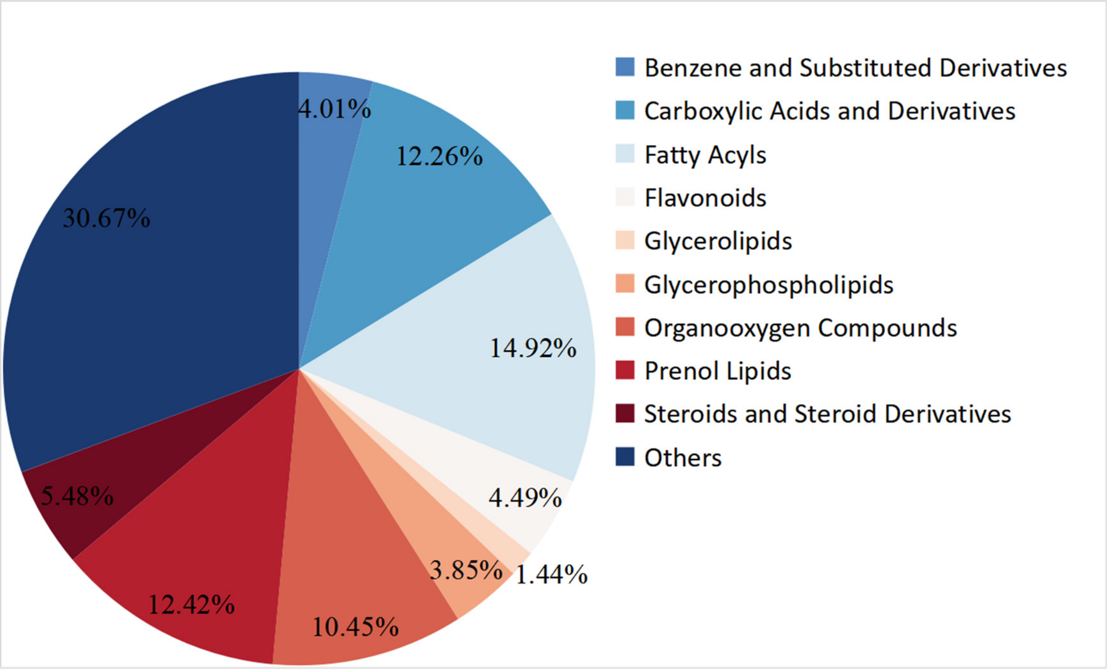

Figure S1. Pie Chart of Metabolite Class Distribution. A quantitative analysis of all metabolite classes within the data matrix was performed. The top 9 most abundant categories were displayed individually, while the remaining classes were aggregated into the "Others" category.

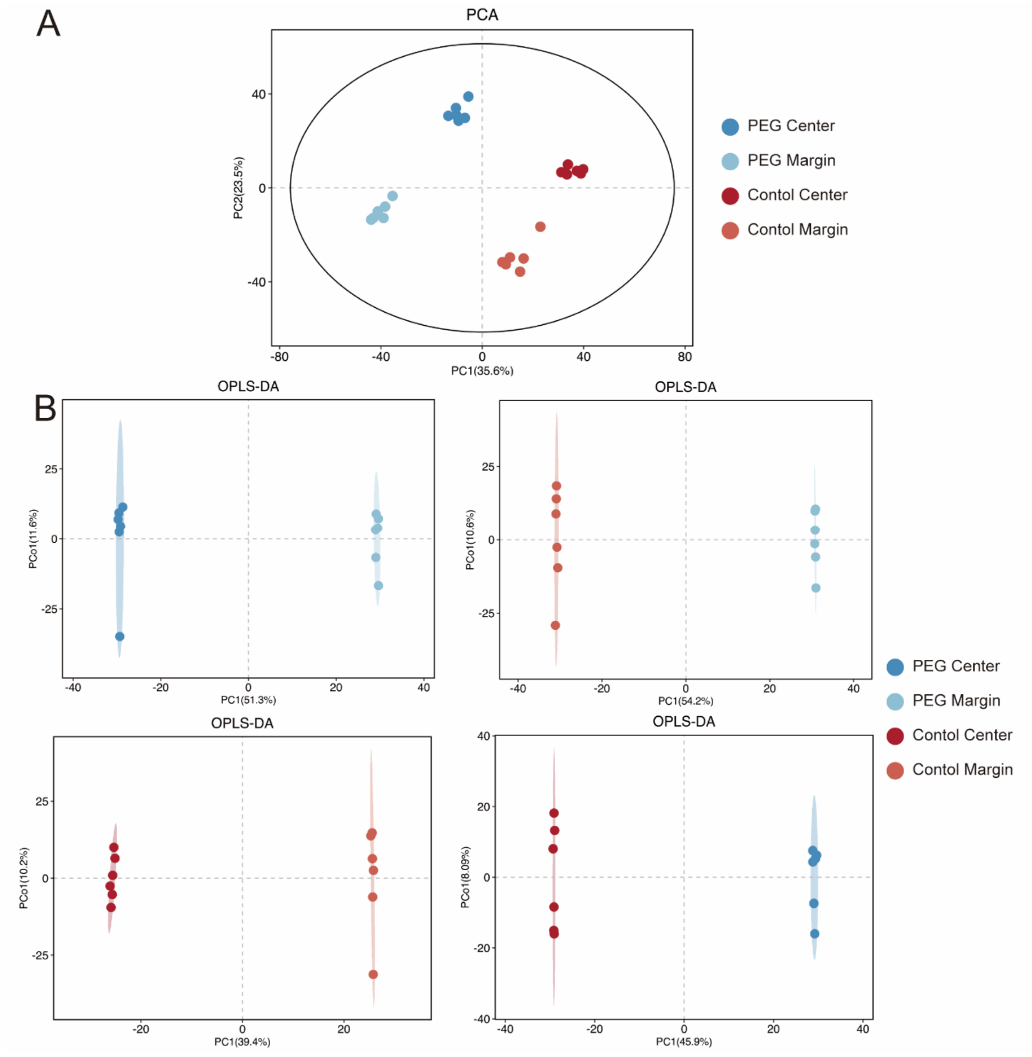

Figure S2. Principal Component Analysis (PCA) and Orthogonal Projections to Latent Structures Discriminant Analysis (OPLS-DA); (A) PCA Plot. The horizontal axis (PC1) represents the first principal component, and the vertical axis (PC2) represents the second principal component. Numbers in parentheses indicate the corresponding variance explained (explanatory rate). Each point corresponds to an individual sample, with different colors denoting distinct sample groups. (B) OPLS-DA. PC1 (Predictive Component): Represents the primary component that maximizes between-group differences (discriminatory variance). PCo1 (Orthogonal Component): Reflects within-group variation (non-discriminatory variance). Each point corresponds to an individual sample.

Table S2. Fold Changes and Significance Analysis of TCA Cycle Metabolites

Metabolites	Formula	foldchange	p-value
Citric acid	$C_6H_8O_7$	1.899	0.272
Malic acid	$C_4H_6O_5$	1.361	0.011
Succinic acid	$C_4H_6O_4$	1.381	0.003
Fumaric acid	$C_4H_4O_4$	0.835	0.621
Oxalacetic acid	$C_4H_4O_5$	1.022	0.790

Notes, FC = (PEG_Margin / PEG_Center) / (Control_Margin / Control_Center)

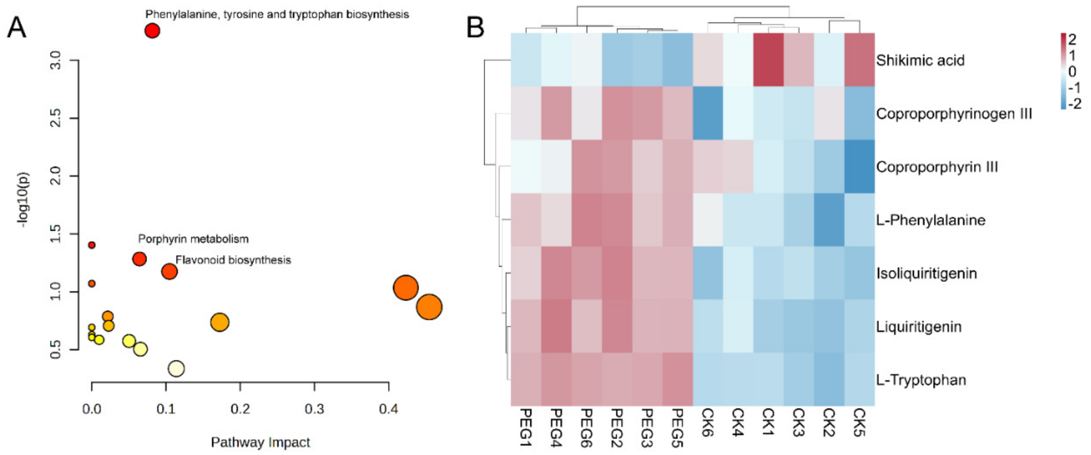

Figure S3. Overview and clustering analysis of pathway-enriched metabolites. (A) Complete set of significantly enriched KEGG pathways. (B) Showing key metabolites from defense-related biosynthesis pathways, based on comparisons within the control group (Control Margin vs. Control Center) and within the drought-stressed group (PEG Margin vs. PEG Center).

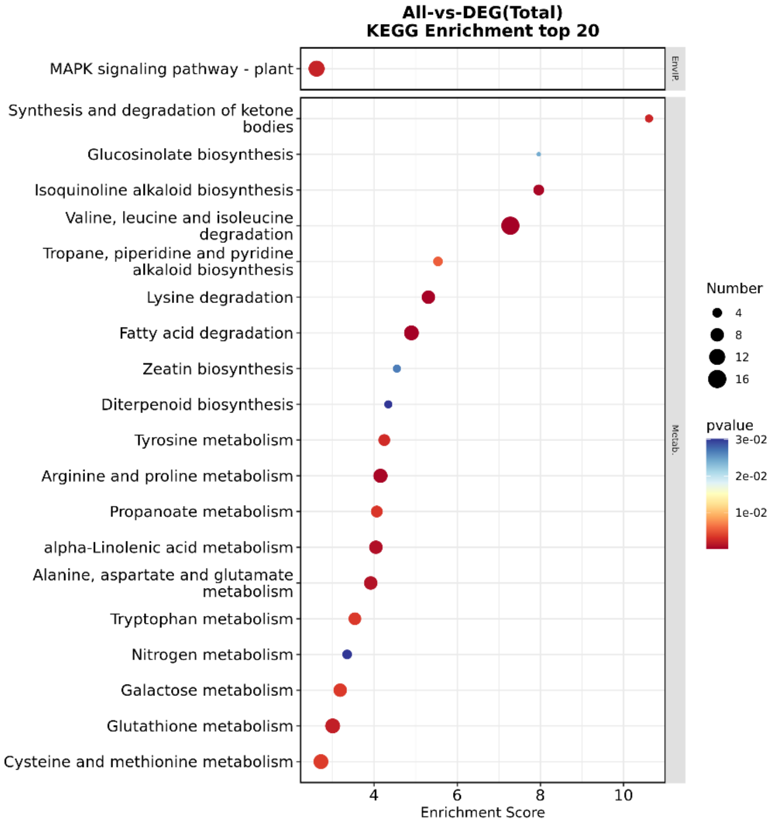

Figure S4. Pathway enrichment analysis of differentially expressed genes. DEGs were screened by comparing Margin vs center zones within both the Control and Drought-stressed

(PEG) groups, applying a fold-change of >2 or <0.5,

Table S3. Changes in Metabolite Levels and Significance Analysis in Defense Metabolic Pathways

Metabolites	Formula	foldchange	p-value
Liquiritigenin	C15H12O4	2.188	2.13E-06
L-Tryptophan	C11H12N2O2	2.696	7.20E-07
L-Phenylalanine	C9H11NO2	2.367	1.61E-03
Isoliquiritigenin	C15H12O4	2.467	3.91E-06
Shikimic acid	C7H10O5	0.489	9.39E-03
Coproporphyrinogen III	C36H44N4O8	3.284	6.69E-03
Coproporphyrin III	C36H38N4O8	2.227	3.82E-02

Notes, FC = (PEG_Margin / PEG_Center) / (Control_Margin / Control_Center)
